# Supplementary material for: Identifying research priorities for pregnant South Asian immigrants in Canada: A James Lind Alliance approach
Source: PLoS One. 2025 Aug 28;20(8):e0330628. doi: 10.1371/journal.pone.0330628 (PMC12393747; doi:10.1371/journal.pone.0330628)
Supplement: S1 File — (PDF) [file pone.0330628.s001.pdf]

S1 File. Semi-structured Guide for Working Group Session with Clinicians and Researchers

Theme 1: Structural Barriers

- What are the key barriers that PSAIs face in accessing timely and appropriate perinatal care in Canada?
- Have you noticed any patterns where cultural or language differences contribute to delays in care or affect care outcomes for PSAIs?
- How do PSAIs experience navigating the healthcare system?
- What role do social factors, such as immigration status, housing insecurity, financial limitations, or lack of social support, play in shaping PSAIs' access to perinatal care?
- Can you share a real-world situation or example that highlights how one or more of these structural barriers affected a PSAI's pregnancy journey?

Theme 2: Research Priorities

- What do you consider the most urgent research priorities to improve health and well-being outcomes for PSAIs?
- From your clinical experience, do current care models adequately meet the needs of PSAIs? Where do they fall short?
- Are there specific tools, screening processes, or models of care that you believe should be culturally adapted to better serve PSAIs?
- What are the most pressing knowledge gaps that limit our ability to provide culturally responsive care to PSAIs?

Theme 3: Collaboration with Community Organizations and Patient Partners

- How can clinicians, researchers, patient partners, and community organizations collaborate more closely to support PSAIs?
- What are some challenges you have experienced (or anticipate) when trying to collaborate with community and/or patient partners in this space?
- Can you share any examples where collaboration among clinicians, researchers, patients and community partners led to better outcomes for immigrant populations, including PSAIs?
- What kinds of support (e.g., resources, infrastructure, training) would help you or your organization engage more meaningfully in partnerships with community and patient partners to improve care for PSAIs?

Theme 4: Next Steps: Future Grant Planning

- What opportunities do you see for multi-site or interdisciplinary grant applications focused on PSAIs?
- Are there particular funding streams (e.g., CIHR, SSHRC, provincial) you recommend we explore for future work in this area?
- What roles (e.g., Co-Applicant, Knowledge User, Collaborator, Implementation Partner) would you be willing to take on?
- Are there specific areas (e.g., needs assessments, co-design of culturally tailored interventions, implementation science, digital health evaluation, or policy translation) where further research is urgently needed to support PSAIs?
- What steps should we prioritize in the next 6–12 months to strengthen our readiness for a competitive, high-impact grant application?
