# Supplementary material for: Identifying research priorities for pregnant South Asian immigrants in Canada: A James Lind Alliance approach
Source: PLoS One. 2025 Aug 28;20(8):e0330628. doi: 10.1371/journal.pone.0330628 (PMC12393747; doi:10.1371/journal.pone.0330628)
Supplement: S2 File — (PDF) [file pone.0330628.s002.pdf]

S2 File. Semi-structured Guide for Working Group Session with Patient Partners

Theme 1: Lived Experience of Pregnancy in Canada

- Can you tell me about your experience of being pregnant in Canada?
- What were the positive aspects of your pregnancy care in Canada?
- What were some of the challenges or difficulties you faced?
- How did your experience here compare to what you expected, or to experiences in your home country?

Theme 2: Accessing the Healthcare System

- How easy or difficult was it for you to find and access prenatal care? Walk us through your journey!
- Were there any challenges with booking appointments, transportation, or costs?
- Did you feel that the healthcare providers understood your needs and concerns?
- Were you ever offered interpreter services or culturally relevant resources?
- What was missing in the care you received during or after your pregnancy?

Theme 3: Social Support and Wellbeing

- Who supported you the most during your pregnancy (e.g., partner, family, friends, community)?
- Did you ever feel lonely or isolated during your pregnancy?
- Were you aware of or connected to any community or social support programs/social services?
- Did you feel comfortable talking about emotional or mental health concerns with your provider?
- What was missing in terms of social services and support systems during your pregnancy?

Theme 4: Cultural Relevance and Respect in Care

- Did you feel that your cultural practices or beliefs were respected during pregnancy or childbirth?
- Were there any cultural or religious needs that were not met by the healthcare system?
- Did you experience any form of discrimination or stereotyping from healthcare providers?

Theme 5: Information, Communication, and Education

- Did you feel well-informed about your pregnancy and what to expect?
- Was information provided in a language you were comfortable with?
- Were you able to ask questions or share your preferences with your provider?
- What type of information or education would have helped you more?

Theme 6: Research Priorities and Needs

- What are the biggest issues or challenges that you think pregnant South Asian women in Canada face?
- What kind of support or programs do you think would help women like you?
- If you could suggest/guide future work in this area, what questions or topics would you prioritize to support the health and well-being of pregnant South Asian immigrants like you?
- How can you (as patient partners) work closely with researchers, community organizations, and clinicians to support pregnant South Asian immigrants?
- If you could change one thing about the pregnancy experience for South Asian immigrants, what would it be?
