# Supplementary material for: Identifying research priorities for pregnant South Asian immigrants in Canada: A James Lind Alliance approach
Source: PLoS One. 2025 Aug 28;20(8):e0330628. doi: 10.1371/journal.pone.0330628 (PMC12393747; doi:10.1371/journal.pone.0330628)
Supplement: S3 File — (PDF) [file pone.0330628.s003.pdf]

S3 File. Semi-structured Guide for Working Group Session with Community Organizations

Theme 1: Structural Barriers and System-Level Support for PSAIs

- What are the main barriers pregnant South Asian immigrants face in accessing or receiving perinatal care in Canada?
- What health and social services are currently available to pregnant South Asian immigrants? How impactful are these existing services?
- From your organization's perspective, what challenges do you face in supporting pregnant South Asian immigrants in Canada?
- What role do social factors, such as immigration status, housing insecurity, financial limitations, or lack of social support, play in shaping PSAIs' access to perinatal care?
- What is missing in terms of services, support systems, training, or policies to effectively meet the needs of pregnant South Asian immigrants?
- Can you share a real-world situation or example that highlights how one or more of these structural barriers affected a PSAI's pregnancy journey?

Theme 2: Research Priorities

- What do you consider the most urgent research priorities to improve health and well-being outcomes for PSAIs?
- Clinical priorities related to common and high-risk pregnancy complications (e.g., GDM, hypertensive disorders, anemia).
- Gaps in clinical care across the pregnancy continuum — preconception, antenatal, intrapartum, and postpartum.
- Cultural responsiveness and inclusivity of the current perinatal care landscape.
- Language and communication barriers in clinical interactions.
- Effectiveness of current screening tools and clinical guidelines for PSAIs.
- Training needs of healthcare providers to deliver culturally safe care.
- Role of digital tools in improving clinical outcomes.

Theme 3: Collaboration with Community Organizations and Patient Partners

- How can clinicians, researchers, patient partners, and community organizations collaborate more closely to support PSAIs?
- What are some challenges you have experienced (or anticipate) when trying to collaborate with community and/or patient partners in this space?
- Can you share any examples where collaboration among clinicians, researchers, patients and community partners led to better outcomes for immigrant populations, including PSAIs?
- What kinds of support (e.g., resources, infrastructure, training) would help you or your organization engage more meaningfully in partnerships with community and patient partners to improve care for PSAIs?

Theme 4: Next Steps: Future Grant Planning

- What opportunities do you see for multi-site or interdisciplinary grant applications focused on PSAIs?
- Are there particular funding sources you would recommend exploring for future work in this area?
- What roles (e.g., Co-Applicant, Knowledge User, Collaborator) would you be willing to take on?
- Are there specific areas (e.g., needs assessments, co-design of culturally tailored interventions, implementation science, digital health evaluation, or policy translation) where further research is urgently needed to support PSAIs?
- What steps should we prioritize in the next 6–12 months to strengthen our readiness for a competitive, high-impact grant application?
