# Supplementary material for: Identifying research priorities for pregnant South Asian immigrants in Canada: A James Lind Alliance approach
Source: PLoS One. 2025 Aug 28;20(8):e0330628. doi: 10.1371/journal.pone.0330628 (PMC12393747; doi:10.1371/journal.pone.0330628)
Supplement: Attachment — Submitted filename: Plos Revi.docx [file pone.0330628.s004.docx]

**Introduction**

1. The Introduction effectively outlines the broader issue of inadequate prenatal care for immigrants and PSAIs. However, the central research gap—the lack of prioritized research on PSAIs’ unique needs—could be stated more clearly and earlier. Consider introducing the research gap in the first 2–3 paragraphs to orient the reader.
2. The term "Pregnant South Asian Immigrants (PSAI)" is introduced midway through the Introduction. Consider defining this term earlier and using it consistently throughout the section to improve readability.
3. Ensure clarity when referring to "immigrants," "newcomers," and "racialized groups" to avoid conflating different populations. Precision in language will aid in scholarly accuracy.
4. The use of references is comprehensive and relevant. However, some references are clustered in long numeric chains (e.g., 4–7; 8–13), which can obscure individual contributions. If feasible, highlight one or two key studies to provide depth rather than breadth.
5. The manuscript mentions "clinical observations from clinicians" (line 20) without referencing specific studies or institutional data. Consider supporting this statement with documented clinical insights or remove it to maintain academic rigor.
6. The brief review of nine studies (lines 25–37) is informative but would benefit from clearer synthesis. Presenting the findings thematically (e.g., structural barriers, cultural mismatches, health literacy) could help the reader better grasp the major barriers faced by PSAIs.
7. While demographic data about South Asians in Canada is helpful, the rationale for focusing specifically on this group (as opposed to other immigrant populations) could be strengthened. Are there known differences in outcomes or health system interactions compared to other immigrant groups?

**Material & Methods:**

1. While the JLA framework is briefly justified (as “inclusive” and “partnership-driven”), further elaboration on why it is specifically suitable for this cultural and immigrant context would enhance the rationale. Has the JLA model been previously applied with immigrant or minority populations in Canada or internationally?
2. The timeline is clearly stated, but the narrative format could benefit from a table or figure to illustrate phases, activities, and responsible parties. This would help readers visualize the project structure and timelines.
3. The section mentions “South Asian immigrant women with pregnancy experience in Canada within the last 5 years” but does not specify further eligibility criteria (e.g., age range, language proficiency, immigration status). Clarifying these parameters would improve replicability and transparency.
4. It's unclear whether inclusion is limited to cisgender women or if the study is inclusive of gender-diverse individuals. This should be explicitly stated.
5. Although the authors rightly state that formal sample size calculations are not needed for JLA projects, it would be helpful to discuss how diversity will be ensured across subgroups (e.g., different South Asian communities, provinces, socioeconomic status).
6. Since many participants are being recruited through existing relationships, a brief discussion of potential selection bias and how it will be mitigated would be appropriate.
7. The use of semi-structured guides and Zoom-based sessions is appropriate and well-justified given geographic dispersion. However, it would be useful to include an example topic or question from the guide in an appendix or supplementary material to increase transparency.
8. You mention “content analysis” but do not explain the approach (e.g., inductive or deductive coding, use of software like NVivo, coder training, inter-rater reliability checks). This should be described more fully, even if analysis is limited to sorting and categorizing uncertainties.
9. Informed consent and honoraria for participants are well addressed. However, there is no mention of ethics approval (e.g., institutional review board/ethics committee) or whether it is in progress. This should be explicitly stated.
10. The plan for emotional support during the final workshop is commendable. Consider briefly describing what qualifications or training the support person will have.
11. he ranking and scoring methodology during the interim phase could benefit from clarification. Will participants rank all priorities or select a subset? Will average scores or a specific consensus threshold determine advancement?
12. For the final workshop, more information on how consensus will be operationalized (e.g., nominal group technique, majority vote) would strengthen methodological clarity.
13. he text occasionally shifts between future and past tense (e.g., "a committee was formed" vs. "participants will be invited"). Consistency in verb tense would improve readability.
14. The phrase “refining complex suggestions into clearly defined research questions” could be clearer if you mention whether these will be phrased using frameworks like PICO or similar.
15. Consider citing one or two recent JLA-based studies in perinatal or immigrant health to show alignment with best practices.
