# Supplementary material for: Identifying research priorities for pregnant South Asian immigrants in Canada: A James Lind Alliance approach
Source: PLoS One. 2025 Aug 28;20(8):e0330628. doi: 10.1371/journal.pone.0330628 (PMC12393747; doi:10.1371/journal.pone.0330628)
Supplement: Attachment — Submitted filename: PONE-D-25-29272_reviewer.docx [file pone.0330628.s005.docx]

|  | Reviewer comment |
| --- | --- |
| Title | **Strength** :  -Clear ,and descriptive, effectively state the study focus and targeted population , it specifies the methodological approach, using protocol clarify that it is methodology plan not result  **Suggested improvements:**  -It is long title ( 22 words) which may affect readability.  -Scope may be too broad as south Asian immigrants include diverse ethnicity, religion ,languages with varying health needs , it may be better if study focus on specific subgroup or acknowledge this as limitation of the study |
| Abstract | **Strength** :   - Clear and strong justification - Knowledge gap is well articulated , focus on lack of culturally appropriate care - Robust methodology , using the setting and identifying the stakeholders enhance the study relevance and credibility - Ethical approval is recognized   **Areas of improvements :**  -Deficiency of description in methods, no detail on the content analysis, how survey will be collected, how consensus will be reached, is the subgroup diversity will be inspected.  - the time to conduct the study is it enough  -Background need readability improvements |
| Ethic statement | **Strength** :  -Clear documentation of approval  - Clear comprehensive informed consent  Clear date protection, restriction to researchers and maintain confidentiality  -Offering compensation for participants  **Areas of improvements**  -Lack of details on vulnerable population protection process and what constitutes vulnerable population  -How long data will be retained and method of storage ,plans for final data destruction  - how consent will be obtain from non-English speakers |
| Introduction | Strength:   - Established global significance effectively ,creating strong rational for focusing on south Asian immigrants - Provides well demographic data about immigrant pattern in Canada - Clearly stated high risks faced by immigrant in comparison to other ethnic group - Clearly identify knowledge gap via literature review that researches are limited - Well-structured and logically flow   **Areas of improvements**  -Differentiate subgroups ,their diversity  -Does not mention existing Canadian policies or program addressing immigrants maternal health  -Search methodology not clear  - Integrate framework to strengthen the study analytical foundation |
| Material and Methods: | |
| Design | **Strength :**   - Appropriate selection of Methodology - Clear structured process with five steps - Emphasis on partnership of stakeholder, and adaptability to immigrant health care   **Areas of improvements:**  -Although JIA’s cited as wide-ranging and comprehensive, there is lack of how cultural, language barriers will be addressed  - Limited data analysis discussion  -Address selection bias |
|  |  |

Dear Editor,

Thank you for the opportunity to review this manuscript. I appreciate the chance to contribute to the evaluation of this work. Below you will find my detailed assessment, which I hope will be helpful in your editorial decision making process .
