# Supplementary material for: Identifying research priorities for pregnant South Asian immigrants in Canada: A James Lind Alliance approach
Source: PLoS One. 2025 Aug 28;20(8):e0330628. doi: 10.1371/journal.pone.0330628 (PMC12393747; doi:10.1371/journal.pone.0330628)
Supplement: Attachment — Submitted filename: Response to Reviewers.docx [file pone.0330628.s007.docx]

**COVER LETTER**

**Manuscript ID: PONE-D-25-29272**

Title: Identifying Research Priorities for Pregnant South Asian Immigrants in Canada: A James Lind Alliance Approach

Dear Dr. Ahmad H. Al-Nawafleh,

Thank you for the opportunity to revise and resubmit our manuscript to PLOS ONE. We are grateful for the thoughtful comments and constructive feedback provided by the editor and reviewers. We have carefully considered all suggestions and revised the manuscript accordingly.

Please find enclosed a detailed point-by-point response to the editor and reviewers’ comments in the table below.

As requested, we have included the following statement regarding the role of the funder in our study: ***“The funders had no role in study design, data collection and analysis, decision to publish, or preparation of the manuscript.”***

We appreciate your consideration of our manuscript and look forward to the possibility of its publication in PLOS ONE.

Sincerely,

Anam Shahil-Feroz (she/her)
Assistant Professor
Arthur Labatt Family School of Nursing, Faculty of Health Sciences
FIMS & Nursing Building, Room 3338
Western University, London, ON, Canada N6A 5B9
<https://www.uwo.ca/fhs/nursing/about/faculty/research_supervisors/anam_s.html>

Journal Requirements

| S. No. | Comment | Point by point response |
| --- | --- | --- |
| 1. | Please ensure that your manuscript meets PLOS ONE's style requirements, including those for file naming. The PLOS ONE style templates can be found at  <https://journals.plos.org/plosone/s/file?id=wjVg/PLOSOne_formatting_sample_main_body.pdf> and  [https://journals.plos.org/plosone/s/file?id=ba62/PLOSOne_formatting_sample_title_authors_affiliations.pdf](https://track.editorialmanager.com/CL0/https:%2F%2Fjournals.plos.org%2Fplosone%2Fs%2Ffile%3Fid=ba62%2FPLOSOne_formatting_sample_title_authors_affiliations.pdf/1/010f01981eac6422-d331b084-5242-4490-add1-82b95550a881-000000/kLVfyeLo3MKO1dMIcd1ZVJ6yunROrkgieu0iz6ZdliI=220) | We have ensured that our manuscript meets *PLOS ONE*'s style requirements. |
| 2. | Please provide additional information regarding the considerations  made for the pregnant South Asian immigrants included in this study. For instance, please discuss whether participants were able to opt out of the study and whether individuals who did not participate receive the same treatment offered to participants. | We have included the ethics statement and clarified that participants, including pregnant South Asian immigrants, are able to opt out of the study without any consequences. |
| 3. | Thank you for stating the following financial disclosure: [This research is funded by the Canadian Institutes of Health Research Planning and Dissemination Grant (Application No. 197315).].  Please state what role the funders took in the study.  If the funders had no role, please state: ""The funders had no role in study design, data collection and analysis, decision to publish, or preparation of the manuscript.""  If this statement is not correct you must amend it as needed.  Please include this amended Role of Funder statement in your cover letter; we will change the online submission form on your behalf. | We have revised the funding statement to include: *“The funders had no role in study design, data collection and analysis, decision to publish, or preparation of the manuscript.”* |
| 4. | When completing the data availability statement of the submission form, you indicated that you will make your data available on acceptance. We strongly recommend all authors decide on a data sharing plan before acceptance, as the process can be lengthy and hold up publication timelines. Please note that, though access restrictions are acceptable now, your entire data will need to be made freely accessible if your manuscript is accepted for publication. This policy applies to all data except where public deposition would breach compliance with the protocol approved by your research ethics board. If you are unable to adhere to our open data policy, please kindly revise your statement to explain your reasoning and we will seek the editor's input on an exemption. Please be assured that, once you have provided your new statement, the assessment of your exemption will not hold up the peer review process. | We have included a data availability statement noting that this is a study protocol, and therefore, no datasets were generated or analyzed during the current study. |
| 5. | Your ethics statement should only appear in the Methods section of your manuscript. If your ethics statement is written in any section besides the Methods, please delete it from any other section. | We have moved the ethics statement to the Methods section only and removed it from the Abstract. |
| 6. | If the reviewer comments include a recommendation to cite specific previously published works, please review and evaluate these publications to determine whether they are relevant and should be cited. There is no requirement to cite these works unless the editor has indicated otherwise. | We appreciate the reviewers’ comments and have carefully reviewed and evaluated their suggestions before incorporating them into the final manuscript. |
| 7. | Please review your reference list to ensure that it is complete and correct. If you have cited papers that have been retracted, please include the rationale for doing so in the manuscript text, or remove these references and replace them with relevant current references. Any changes to the reference list should be mentioned in the rebuttal letter that accompanies your revised manuscript. If you need to cite a retracted article, indicate the article’s retracted status in the References list and also include a citation and full reference for the retraction notice. | We have reviewed our reference list to ensure that it is complete and accurate. |

EDITOR COMMENTS

| S. No. | Comment | Point by point response |
| --- | --- | --- |
| 1. | Probably you need to revise the title too | We have revised the title, and the new suggested title is:  Identifying Research Priorities for Pregnant South Asian Immigrants in Canada: A James Lind Alliance Approach |

REVIEWER 1 COMMENTS

| 1. | Title: It is long title ( 22 words) which may affect readability.  -Scope may be too broad as south Asian immigrants include diverse ethnicity, religion ,languages with varying health needs , it may be better if study focus on specific subgroup or acknowledge this as limitation of the study | We have revised the title for readability, and the new suggested title is:  Identifying Research Priorities for Pregnant South Asian Immigrants in Canada: A James Lind Alliance Approach.  Regarding the scope of the study, we recognize that the term “South Asian immigrants” encompasses a highly diverse group with varied cultural, linguistic, and health-related experiences. While we acknowledge this diversity, our intent was to use a more inclusive framing to reflect the collective gaps in perinatal research that many South Asian immigrant populations share in Canada. However, to maintain feasibility and contextual relevance, we focused specifically on first-generation South Asian immigrant women from Bangladesh, India, Pakistan, and Sri Lanka who are currently pregnant or have recently experienced pregnancy in Canada.  We have now clearly described this focus in the manuscript and explicitly acknowledged the broader use of “South Asian” as a limitation in the Limitations section. |
| --- | --- | --- |
| 2. | Abstract: Deficiency of description in methods, no detail on the content analysis, how survey will be collected, how consensus will be reached, is the subgroup diversity will be inspected.  - the time to conduct the study is it enough  Background need readability improvements | We have revised the abstract to include greater detail on the methodology, including how data will be collected (Zoom sessions and surveys), how content analysis will be conducted (inductive approach), and how consensus will be reached (structured ranking followed by a final workshop). We have also clarified that subgroup diversity will be tracked across roles, ethnicity, gender, and geographic region. In addition, we have improved the readability of the background and added a statement to support the feasibility of the proposed timeline based on similar JLA PSPs. All these changes have been incorporated into the revised abstract. |
| 3. | Ethics Statement: Lack of details on vulnerable population protection process and what constitutes vulnerable population  -How long data will be retained and method of storage ,plans for final data destruction  - how consent will be obtain from non-English speakers | Thank you for this important comment. We have revised the Ethics section of the manuscript to provide additional details as follows:   - We now explicitly identify pregnant South Asian immigrants (PSAIs) as the vulnerable population in this study. - We describe the measures taken to protect this population, including the use of plain language in all consent materials and verbal explanation of consent forms to support understanding. - We now clarify that study data will be stored on encrypted, password-protected servers at Western University, retained for three years or until publication, whichever comes first, and permanently deleted afterward according to institutional data destruction protocols. |
| 4. | Differentiate subgroups ,their diversity  -Does not mention existing Canadian policies or program addressing immigrants maternal health  -Search methodology not clear  - Integrate framework to strengthen the study analytical foundation | As mentioned earlier, we recognize that the term “South Asian immigrants” encompasses a highly diverse group with varied cultural, linguistic, and health-related experiences. While acknowledging this diversity, we adopted an inclusive framing to highlight the shared structural and systemic gaps in perinatal health research that affect many South Asian immigrant populations in Canada. To maintain feasibility and contextual relevance, this study specifically focused on first-generation South Asian immigrant women from Bangladesh, India, Pakistan, and Sri Lanka who are currently pregnant or have recently experienced pregnancy in Canada. We have mentioned this in method’s section.  In response to the reviewer’s comment, we have incorporated additional literature on immigrant maternal health in the Canadian context. However, our preliminary scoping work and review of the literature reveal that there is a significant gap in research specifically focused on South Asian immigrant pregnant populations. Our research team is currently conducting a scoping review to systematically identify and synthesize evidence in this area.  Given the limited availability of relevant literature and the absence of an existing framework specific to this population, we have chosen to take an inductive approach to data analysis. This approach will allow us to derive priorities and themes grounded in the data and reflective of participants’ lived experiences. |
| 5. | Design: Although JIA’s cited as wide-ranging and comprehensive, there is lack of how cultural, language barriers will be addressed  - Limited data analysis discussion  -Address selection bias | Thank you for your comments. We have revised the manuscript to address the concerns raised.  1. Cultural and Language Barriers: We have elaborated on how cultural and language barriers will be addressed in strengths and limitations section.  2. Data Analysis: We have expanded the data analysis section to detail our inductive analysis approach.  3. Selection Bias: We now explicitly acknowledge the potential for selection bias and describe strategies to minimize its impact. See the limitation section. |

REVIEWER 2 COMMENTS

| 1. | Introduction:  The Introduction effectively outlines the broader issue of inadequate prenatal care for immigrants and PSAIs. However, the central research gap—the lack of prioritized research on PSAIs’ unique needs—could be stated more clearly and earlier. Consider introducing the research gap in the first 2–3 paragraphs to orient the reader. | In response to the suggestion to clarify the central research gap earlier in the introduction, we have revised the first paragraph to more clearly and explicitly state the lack of research and prioritization regarding the unique perinatal care needs of pregnant South Asian immigrants (PSAIs) in Canada. |
| --- | --- | --- |
| 2. | The term "Pregnant South Asian Immigrants (PSAI)" is introduced midway through the Introduction. Consider defining this term earlier and using it consistently throughout the section to improve readability. | Thank you for this suggestion. We have now defined the term "Pregnant South Asian Immigrants (PSAI)" at the beginning of the Introduction and ensured that it is used consistently throughout the section to enhance clarity and readability. |
| 3. | Ensure clarity when referring to "immigrants," "newcomers," and "racialized groups" to avoid conflating different populations. Precision in language will aid in scholarly accuracy. | Thank you for your suggestion. To improve clarity and consistency, we have now used the term **“immigrants”** exclusively throughout the manuscript and have removed the terms **“racialized groups”** and **“newcomers.”** |
| 4. | The use of references is comprehensive and relevant. However, some references are clustered in long numeric chains (e.g., 4–7; 8–13), which can obscure individual contributions. If feasible, highlight one or two key studies to provide depth rather than breadth. | Thank you for the suggestion. We have revised the manuscript to highlight key barriers faced by pregnant South Asian immigrant women in Canada, including language difficulties, low health literacy, acculturative stress, financial challenges, limited access to culturally appropriate care, and higher risks of gestational diabetes and perinatal depression. This provides a clearer understanding of their unique healthcare needs. |
| 5. | The manuscript mentions "clinical observations from clinicians" (line 20) without referencing specific studies or institutional data. Consider supporting this statement with documented clinical insights or remove it to maintain academic rigor. | Thank you for your suggestion. As per your recommendation, we have removed the statement referring to clinical observations from clinicians and instead included references to the small but relevant body of published evidence to support the point. |
| 6. | The brief review of nine studies (lines 25–37) is informative but would benefit from clearer synthesis. Presenting the findings thematically (e.g., structural barriers, cultural mismatches, health literacy) could help the reader better grasp the major barriers faced by PSAIs. | Thank you for this suggestion. In response, we have revised the brief review of the studies (lines 22–34) to present the findings using a thematic structure. Specifically, we have grouped the barriers faced by pregnant South Asian immigrants into the following categories: structural, cultural, communication and informational, and health system trust and responsiveness barriers. We believe this thematic synthesis enhances the clarity, coherence, and accessibility of this section for readers. |
| 7. | While demographic data about South Asians in Canada is helpful, the rationale for focusing specifically on this group (as opposed to other immigrant populations) could be strengthened. Are there known differences in outcomes or health system interactions compared to other immigrant groups? | Thank you for this comment. I have strengthened the rationale for focusing specifically on South Asian immigrant pregnant individuals by highlighting evidence that this group experiences higher rates of adverse pregnancy outcomes such as gestational diabetes and preeclampsia compared to other immigrant groups. I have also elaborated on contributing factors that uniquely affect this population, including restrictive gender norms, stigma, language barriers, and a lack of culturally and linguistically appropriate services. |
| 1. | Material and Methods:  While the JLA framework is briefly justified (as “inclusive” and “partnership-driven”), further elaboration on why it is specifically suitable for this cultural and immigrant context would enhance the rationale. Has the JLA model been previously applied with immigrant or minority populations in Canada or internationally? | While the James Lind Alliance (JLA) Priority Setting Partnership methodology has not yet been applied specifically to immigrant populations, it has been widely used to identify research priorities in various health contexts, particularly chronic conditions. In our CIHR Planning Grant application, we proposed adapting the JLA approach to identify priority perinatal care needs among immigrant communities, an approach that was well-received by peer reviewers. We have articulated in conclusion section that JLA approach has not yet been used to identify research priorities specifically for immigrant populations, highlighting the novelty and potential contribution of this study. |
| 2. | The timeline is clearly stated, but the narrative format could benefit from a table or figure to illustrate phases, activities, and responsible parties. This would help readers visualize the project structure and timelines. | Thank you for this suggestion. We have added a timeline table to clearly outline the project phases, key activities, and responsible parties to enhance clarity and help readers visualize the study structure and timelines. |
| 3. | The section mentions “South Asian immigrant women with pregnancy experience in Canada within the last 5 years” but does not specify further eligibility criteria (e.g., age range, language proficiency, immigration status). Clarifying these parameters would improve replicability and transparency. | Thank you for this helpful comment. We have now clarified the eligibility criteria under the Research Setting and Participants section to improve transparency and replicability. |
| 4. | It's unclear whether inclusion is limited to cisgender women or if the study is inclusive of gender-diverse individuals. This should be explicitly stated. | Thank you for pointing this out. We have now clarified in the Research Setting and Participants section that the study includes all individuals assigned female at birth who identify as women or gender-diverse and have experienced pregnancy, to ensure inclusivity. |
| 5. | Although the authors rightly state that formal sample size calculations are not needed for JLA projects, it would be helpful to discuss how diversity will be ensured across subgroups (e.g., different South Asian communities, provinces, socioeconomic status). | Thank you for this important comment. We have addressed this in the Methods and Strengths and Limitations section by describing our purposive sampling strategy to ensure diversity across key characteristics, including immigration status, education, employment, age, parity, and length of time since arrival. |
| 6. | Since many participants are being recruited through existing relationships, a brief discussion of potential selection bias and how it will be mitigated would be appropriate. | We have now included a brief discussion of potential selection bias due to recruitment through existing relationships in the Strengths and Limitations section, along with our strategies to mitigate this by recruiting across multiple provinces and using diverse channels. |
| 7. | The use of semi-structured guides and Zoom-based sessions is appropriate and well-justified given geographic dispersion. However, it would be useful to include an example topic or question from the guide in an appendix or supplementary material to increase transparency. | Thank you for the helpful suggestion. We have added an example of the semi-structured interview guide as supplementary material to increase transparency. |
| 8. | You mention “content analysis” but do not explain the approach (e.g., inductive or deductive coding, use of software like NVivo, coder training, inter-rater reliability checks). This should be described more fully, even if analysis is limited to sorting and categorizing uncertainties. | We have expanded the Data Analysis section to provide more detail on our inductive content analysis approach and included the use of NVivo software to support data management and coding. |
| 9. | Informed consent and honoraria for participants are well addressed. However, there is no mention of ethics approval (e.g., institutional review board/ethics committee) or whether it is in progress. This should be explicitly stated. | We have obtained ethics approval for the study, and this is now explicitly stated under the Ethics heading in the manuscript. |
| 10. | The plan for emotional support during the final workshop is commendable. Consider briefly describing what qualifications or training the support person will have. | We have now included a brief description of the support person's qualifications in the manuscript. Specifically, we note that the support person will be a researcher with experience in trauma-informed care and culturally responsive counselling, particularly with immigrant and racialized populations. |
| 11. | The ranking and scoring methodology during the interim phase could benefit from clarification. Will participants rank all priorities or select a subset? Will average scores or a specific consensus threshold determine advancement? | We have clarified the interim ranking and scoring methodology in the manuscript. Specifically, participants will be asked to rank their top 10 priorities from the long list, and aggregated scores will be used to select the top 15–18 priorities to advance to the final workshop. |
| 12. | For the final workshop, more information on how consensus will be operationalized (e.g., nominal group technique, majority vote) would strengthen methodological clarity. | We have now added further detail on how consensus will be reached during the final workshop. Specifically, we will use a modified nominal group technique, where participants will engage in facilitated discussions, followed by individual ranking and group reflection to reach consensus on the top ten research priorities. |
| 13. | The text occasionally shifts between future and past tense (e.g., "a committee was formed" vs. "participants will be invited"). Consistency in verb tense would improve readability. | The use of past tense in Step 1 (e.g., “a committee was formed”) reflects activities that have already been completed, whereas the future tense in the subsequent steps reflects planned activities that are yet to take place. |
| 14. | The phrase “refining complex suggestions into clearly defined research questions” could be clearer if you mention whether these will be phrased using frameworks like PICO or similar. | We’ve clarified that complex suggestions will be refined into clear research questions using frameworks like PICO where appropriate. |
| 15. | Consider citing one or two recent JLA-based studies in perinatal or immigrant health to show alignment with best practices. | We have now cited recent studies that have applied the JLA methodology to identify research priorities related to pregnancy-associated complications (e.g., gestational diabetes, preeclampsia). However, we were unable to locate JLA-based studies focused specifically on immigrant populations in the perinatal context. This highlights the novelty of our study in applying the JLA approach to engage immigrant communities, particularly South Asian immigrants, in setting priorities related to pregnancy and settlement experiences. |

REVIEWER 3 COMMENTS

| 1. | Introduction:  The literature review could be more comprehensive. Expand on the existing literature regarding maternal health disparities among PSAIs. Consider citing more recent Canadian studies or reports. Include more recent literature on immigrant maternal health outcomes and their integration into healthcare systems. | Thank you for this valuable suggestion. We have expanded the literature review in the Introduction to provide a more comprehensive overview of maternal health disparities among pregnant South Asian immigrants (PSAIs) in Canada. |
| --- | --- | --- |
| 2. | Methods:  While the lack of a formal sample size calculation aligns with JLA methods, providing a brief justification or referencing similar studies could enhance methodological clarity. Clarify how funders and policymakers will be engaged post-dissemination to ensure the uptake of research priorities into practice or policy. | Thank you for your comment. We have added a justification and referenced similar studies in the Methods section regarding the lack of formal sample size calculation in JLA methodology.  In the Conclusion section, we have expanded on strategies to share the findings with various stakeholder groups. In particular, we will develop policy briefs and presentations for decision-makers and funders. Additionally, decision-makers and funders will be invited to participate in stakeholder advisory groups or roundtable discussions to facilitate ongoing dialogue and collaborative planning. |
| 3. | Conclusion  Emphasize the potential policy implications and the scalability of the JLA approach to other immigrant communities. | Thank you for this helpful suggestion. We have revised the Conclusion section to emphasize the potential policy implications of the research priorities identified through this work. We have also highlighted the broader applicability and scalability of the JLA approach to other immigrant communities and marginalized populations. |
